# Climate Change and Emerging Arboviral Threats in Saudi Arabia: Epidemiology, Vector Ecology, and One Health Preparedness

**DOI:** 10.3390/idr18030057

**Published:** 2026-06-12

**Authors:** Shuaibu Abdullahi Hudu, Emad A. Morad, Ghusun M. Alhazimi, Abdulgafar Olayiwola Jimoh

**Affiliations:** 1 Center for Health Research, Northern Border University, Arar 91431, Saudi Arabia; 2 Department of Microbiology, Faculty of Medicine, Northern Border University, Arar 91431, Saudi Arabia; 3 Department of Microbiology, Faculty of Medicine, Zagazig University, Zagazig 7120001, Al-Sharqia Governorate, Egypt; emadmorad2010@gmail.com; 4 Department of Bacteriology Lab, Northern Border Regional Laboratory, Northern Borders Cluster, Arar 91431, Saudi Arabia; ghosonn1@gmail.com; 5 Department of Pharmacology and Therapeutics, Faculty of Basic Clinical Sciences, College of Health Sciences, Usmanu Danfodiyo University, Sokoto 840232, Sokoto State, Nigeria; jimoh.abdulgafar@udusok.edu.ng

**Keywords:** *Aedes aegypti*, arbovirus, climate change, dengue, Hajj, public health preparedness, Saudi Arabia, surveillance, vector control

## Abstract

Arboviral diseases are emerging as important public health threats in Saudi Arabia, driven by rapid urbanization, climate variability, the expansion of *Aedes aegypti* populations, international travel, and large-scale religious mass gatherings. Dengue virus remains the most established arboviral infection in the Kingdom, particularly in the southwestern regions such as Jazan and the western urban centers of Makkah and Jeddah, where ecological and climatic conditions are conducive to sustained vector survival and transmission. This review synthesizes current evidence on the epidemiology, vector ecology, climatic determinants, diagnostics, and prevention strategies of arboviral diseases in Saudi Arabia. Particular attention is paid to the impacts of rising temperatures, changes in rainfall patterns, urban heat island effects, population mobility, and cross-border movement on vector expansion and disease emergence. The review also identifies gaps in surveillance, diagnostics, insecticide resistance monitoring, and integrated vector management programs. Emerging preparedness strategies include climate-informed early warning systems, Geographic Information System-based risk mapping, multiplex molecular diagnostics, genomic surveillance, and community-based vector control. The review emphasizes the importance of implementing a One Health approach that combines data on humans, the environment, entomology, and climate. Currently, sustained endemic transmission of chikungunya and Zika viruses has not been conclusively demonstrated in Saudi Arabia, but increased environmental suitability and connectivity with other areas highlight the need for proactive surveillance and preparedness.

## 1. Introduction

The global burden of arthropod-borne viral diseases has increased substantially during the 21st century, driven in part by the geographic expansion of vector species into previously unaffected regions [1]. This increase and geographic spread are not coincidental but reflect a combination of formidable anthropogenic and environmental determinants. Rapid urbanization, poorly planned redevelopment of farmland or forests, frequent urban flooding, and haphazard city growth create crowded human habitats in squalid conditions, with unreliable water supplies and storage practices that create abundant breeding sites for mosquito vectors [2,3]. Increasing levels of international travel, migration, trade, and mass gatherings facilitate the rapid movement of pathogens across geographical boundaries [4,5]. Climate change further amplifies vector-borne disease transmission by altering environmental conditions favorable for mosquito survival, vector expansion, and viral replication. Such increases can be further driven by rising temperatures that enhance mosquito development, shorten the extrinsic incubation period for viruses, and extend vector ranges into areas previously non-permissive to vectors, as well as by alterations in precipitation patterns that create episodic conditions favoring vector population growth [6,7]. Aedes mosquitoes, most notably *Aedes aegypti*, pose significant arboviral threats. They transmit viruses such as dengue (DENV), Chikungunya (CHIKV), Zika (ZIKV), and yellow fever (YFV). These viruses cause a broad clinical spectrum, ranging from mild febrile illness to severe hemorrhagic, neurological, and congenital complications. These infections account for hundreds of millions of cases annually and impose substantial socioeconomic burdens worldwide [8,9].

On the global stage, Saudi Arabia offers a unique setting for studying arboviral disease risk due to its climatic conditions, international connectivity, and role as a major destination for religious mass gatherings, whether driven by local or external factors. Combined with ecological conditions conducive to arboviral transmission, recent dengue outbreaks in Jazan suggest increasing transmission intensity in the region [10]. The hot, humid Tihama plain of the region is considered ideal for year-round survival of *Aedes aegypti* [11,12]. Moreover, intermittent water supply and the high use of uncovered containers for water storage also support domestic breeding sites [10,13]. These additional risks are compounded by its international religious significance and by its porous Yemeni border, which facilitates rapid cross-border movement of arboviruses into the Kingdom [14,15]. The endemic status of *Aedes aegypti* in populated areas across Saudi Arabia, along with increasing temperatures and extreme weather events, highlights an urgent, under-acknowledged risk of emerging or re-emerging arboviral disease [16]. Population-level seroprevalence data for chikungunya and Zika viruses in Saudi Arabia remain scarce, limiting current understanding of population immunity and susceptibility. Nevertheless, the established presence of competent Aedes aegypti populations, increasing regional connectivity, and repeated opportunities for viral importation may create conditions conducive to localized transmission should these viruses be introduced [17,18]. Current dengue-focused surveillance systems in Saudi Arabia are therefore vulnerable to delayed detection of the silent circulation of other arboviruses (until major outbreaks occur). Importantly, increasing insecticide resistance in *Aedes aegypti* populations undermines traditional fogging-based vector-control programs [1], underscoring the need for more effective, long-lasting, integrated control options [6]. Accordingly, this review synthesizes available evidence on the epidemiology of arboviral infections in Saudi Arabia, as well as ecological and meteorological drivers that inform onward transmission dynamics and previous implementation efforts for control interventions. We review the implications of international travel and cross-border population movements for the introduction and spread of arboviruses. By integrating concepts across disciplines, including epidemiology, vector ecology, and climate forecasts, this review provides an evidence-based framework to strengthen national preparedness and support resilient, One Health-aligned surveillance and response systems. This narrative review synthesized published literature from PubMed, Scopus, Web of Science, and Google Scholar. Relevant articles on arboviral epidemiology, vector ecology, climate drivers, diagnostics, surveillance, vector control, and One Health preparedness in Saudi Arabia and the Arabian Peninsula were thematically reviewed and integrated.

## 2. Geographic Distribution and Recent Trends

**Figure 1:**
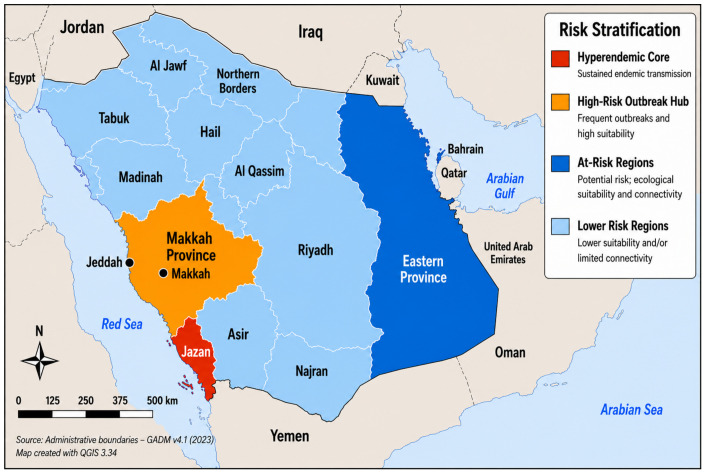
Geographic stratification of arboviral transmission risk in Saudi Arabia, with emphasis on dengue virus transmission. Jazan Province is depicted as the hyperendemic core (red), characterized by sustained endemic transmission and recurrent outbreaks. Makkah Province, including Jeddah, is shown as a high-risk outbreak hub (orange), reflecting repeated dengue outbreaks, high population density, and intense international connectivity associated with Hajj and Umrah pilgrimages. The Eastern Province is categorized as an at-risk region (dark blue) due to ecological suitability and the occurrence of imported and sporadic cases. Remaining provinces are classified as lower-risk regions (light blue), reflecting comparatively lower documented transmission and/or reduced ecological suitability. Administrative boundaries were adapted from GADM v4.1 (2023) geospatial datasets.

The distribution of arboviral infections in Saudi Arabia is not homogeneous, likely due to interactions among environmental factors, vector presence, and human demographics. The main focus of arboviral activity, namely dengue, is in the southwest [19], as shown in Figure 1. Jazan is characterized by low-altitude coastal plains, a hot and humid climate, abundant mosquito breeding habitats, and high population density, all of which favor sustained dengue transmission [20,21]. Jazan outbreaks have been reported since the 1990s, with their frequency, size, and geographical extent increasing tenfold in recent years [22]. Recent epidemiological evidence suggests a transition from sporadic outbreaks to sustained seasonal transmission, particularly during and immediately following the rainy seasons [10,23].

Outside of Jazan, other regions have reported local dengue transmission, indicating a worrisome geographic expansion. Two major global cities for religious tourism are the holiest city, Makkah, and the port city of Jeddah [19,24], both of which were affected by large outbreaks. Additionally, the high population density of those who are neither immune nor vaccinated, together with suboptimal water storage practices in some areas, could lead to explosive outbreaks [25,26]. Similarly, the increased case numbers reported in southern regions are attributed to similar climatic conditions in Jazan and greater travel connectivity [27]. Imported and sporadic cases (within the Eastern Province) have also been reported, often among foreign workers who have returned from endemic areas [21].

Historically, DENV-1 and DENV-2 have been the predominant circulating serotypes in Saudi Arabia. However, recent detection of DENV-3 in regions such as Jazan and Makkah suggests evolving transmission dynamics and ongoing viral introductions. The co-circulation of multiple dengue serotypes is clinically important because secondary infection with a heterologous serotype may increase the risk of severe dengue through antibody-dependent enhancement (ADE), a phenomenon in which non-neutralizing antibodies from a prior infection facilitate viral entry into host cells [27]. Continued molecular surveillance is therefore essential for monitoring serotype shifts and assessing the risk of outbreak severity [28,29]. Documented evidence currently supports dengue endemicity in specific regions of Saudi Arabia, whereas chikungunya and Zika viruses remain potential emerging threats requiring enhanced surveillance rather than confirmed widespread endemic circulation.

## 3. Vector Distribution and Arboviral Transmission Potential

The epidemiology of arboviral diseases in Saudi Arabia is closely linked to the distribution, abundance, and vector competence of mosquito species that transmit human pathogens. Among these, *Aedes aegypti* remains the most important arboviral vector in the Kingdom due to its established role in dengue virus transmission and its wide distribution in urban and peri-urban environments, particularly in Jazan, Makkah, and Jeddah [11,13,30]. This highly anthropophilic mosquito is well adapted to domestic environments, breeding mostly in artificial water-holding containers, including household storage tanks, discarded tires, flowerpots, and air-conditioning drip trays [13,31]. The acquisition of multiple blood meals within a single gonotrophic cycle of the species also increases its vectorial capacity and allows for efficient arbovirus transmission [32,33].

*Aedes albopictus* is also a confirmed vector for several medically important arboviruses, including DENV, CHIKV, and ZIKV worldwide [34]. Although *Aedes albopictus* is not widely established in Saudi Arabia, its gradual geographic expansion in neighboring areas and its remarkable ecological plasticity require continuous entomological surveillance and early detection programs to prevent its establishment and spread in the Kingdom [34]. The introduction of *Aedes albopictus*, which can survive in diverse climatic conditions and exploit both natural and artificial breeding sites, could significantly influence local arboviral transmission dynamics.

Other mosquito genera in Saudi Arabia may also contribute to the emergence of arboviruses under suitable ecological conditions. *Culex* spp. are widely distributed across the Kingdom and have been implicated globally in the transmission of several arboviruses, including West Nile virus and other flaviviruses [16]. Similarly, *Anopheles* spp., although primarily recognized as malaria vectors, are also significant members of the local mosquito fauna and may influence the wider vector-borne disease ecology [20,31]. Their contribution to arboviral transmission in Saudi Arabia currently appears limited, but environmental and climatic changes could affect vector distribution patterns and host–pathogen interactions in the future.

Climate change, rapid urbanization, increased international travel, trade, labor migration, and large-scale religious gatherings all provide favorable conditions for changes in vector distribution, abundance, and seasonal activity [4,5,6,7]. Increasing temperatures and altered precipitation may expand the availability of suitable habitats for mosquito vectors, increase vector survival, facilitate viral replication, and favor the invasion of species into areas where they previously did not occur [6]. These environmental and demographic drivers highlight the need for proactive vector surveillance programs that can detect changes in species composition and geographic distribution prior to outbreaks occurring.

Given the dynamic nature of the arboviral risk in Saudi Arabia, entomological surveillance, accurate species identification, routine insecticide-resistance monitoring, and vector competence studies must be part of national preparedness strategies. Strengthening these activities will enable early detection of emerging threats, support evidence-based vector-control interventions, and increase the country’s capacity to anticipate and respond effectively to future arboviral outbreaks within a One Health framework.

## 4. Vector Ecology and Climate Drivers

The ecology of the primary arbovirus vector, *Aedes aegypti*, is a determinant of transmission dynamics in Saudi Arabia [30]. The highly anthropophilic, day-biting mosquito *Aedes aegypti* is well adapted to urban and peri-urban environments in the Kingdom. Its larvae breed in various artificial water-holding containers, and there are many breeding places in domestic environments. Common larval breeding sites include open tins of water, discarded tires, flower pots, and air-conditioning drip trays [13]. In Jazan, storing water in zeers (clay containers) for intermittent use is a dominant factor in vector breeding [31]. *Aedes aegypti* females frequently take multiple blood meals during a single gonotrophic cycle, increasing opportunities for virus transmission, because a single virus-infected mosquito could transmit the disease to several humans [32,33].

**Figure 2:**
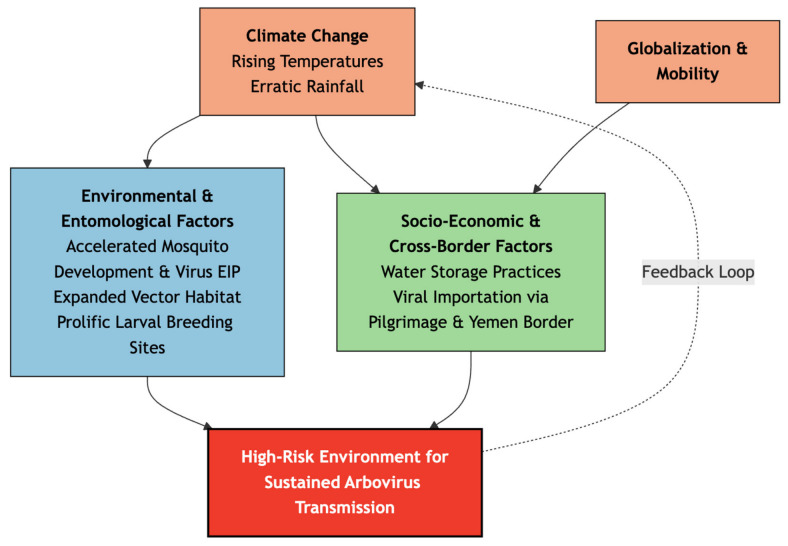
Conceptual framework of climatic, environmental, and socio-demographic drivers of arboviral transmission risk in Saudi Arabia. The figure summarizes the interactions among climate change, urbanization, vector ecology, water-storage practices, and human mobility that influence arboviral transmission dynamics. Climatic factors, including rising temperatures and altered rainfall patterns, may enhance Aedes aegypti survival and transmission potential. Solid arrows indicate hypothesized causal or facilitating pathways through which climatic and socio-environmental factors influence vector ecology and arboviral transmission risk. The curved arrow represents a feedback mechanism whereby increasing transmission risk may further amplify surveillance demands, urban vulnerability, and public health pressures, reinforcing transmission dynamics. The framework highlights the importance of integrated surveillance, climate-informed early warning systems, and One Health preparedness strategies. Created by the authors using BioRender, available online at https://www.biorender.com (accessed on 22 May 2026). and Adobe Illustrator version 28.5, based on mechanisms described in the published literature.

Climate is a key regulator of *Aedes aegypti* population dynamics and thus the risk of arbovirus transmission. Temperature, precipitation, and humidity have both direct and indirect effects. Temperature modulates the life cycle of mosquitoes, such that higher temperatures accelerate larval development, shorten the interval between an infected blood meal and the time at which a mosquito becomes competent to transmit virus, referred to as the extrinsic incubation period (EIP), and increase the adult mosquito biting rate [34,35]. For dengue, the EIP can be 7 days at 30 °C, compared with >15 days at 25 °C, resulting in a significant increase in transmission potential during periods of high temperature [36]. These interconnected factors create a feedback loop of increasing risk, as illustrated in Figure 2.

In Saudi Arabia’s dry landscape, the timing of rainfall is just as important as how much it falls. In areas where water is not consistently available, the seasonality of household behavior and dry-season water storage create year-round breeding sites for a vector capable of maintaining populations year-round [37,38]. Projected increases in temperature and changes in precipitation patterns may enhance environmental suitability for *Aedes aegypti* survival, reproduction, and geographic expansion, although the magnitude of these effects will vary across ecological settings. Strengthening local surveillance systems and regional public-health collaboration will be essential for the early detection and mitigation of emerging arboviral threats. Increasing climatic suitability may facilitate future expansion of vector populations into previously lower-risk regions. A previous study indicated that in high-emission scenarios, the global population exposed to dengue risk may increase as the efficacy of control methods declines, particularly in the Middle East, under a warming, urbanizing climate [39]. The combination of climate and the urban heat island effect in places like Jeddah and Riyadh can create microclimates more conducive to mosquito breeding and viral transmission. To develop predictive models enabling early warning systems, it is essential to understand the intricate non-linear dependence of climate variables on vector-borne disease transmission. The overlap of real-time weather and entomological surveillance data can be leveraged to target high-risk areas and time periods for pre-outbreak vector control.

## 5. Diagnostics and Clinical Spectrum

Accurate diagnosis is an essential prerequisite for controlling arboviral disease, but it is difficult because most of these infections present with vague clinical histories or are non-specific [40,41]. Despite the mild febrile illness more commonly associated with dengue virus infection, severe disease can pose a diagnostic dilemma in Saudi Arabia [42]. In most cases, the patient has an abrupt onset of high fever, severe headache, retro-orbital pain, myalgia (muscle pain), and arthralgia (joint pain). Severe clinical manifestations typically occur during the critical phase of illness, when increased vascular permeability can lead to plasma leakage and hemodynamic instability. This life-threatening event causes the severe manifestations associated with dengue hemorrhagic fever (DHF) and dengue shock syndrome, specifically hemoconcentration, thrombocytopenia, and even fatal cardiovascular collapse [43,44]. Because clinical manifestations range from mild febrile illness to severe life-threatening disease, laboratory confirmation plays a critical role in diagnosis and patient management.

The diagnostic course is tailored to the stage of infection. Monitoring for viral detection or related components is usually employed in the initial acute phase, ideally within 1 to 5 days after symptom onset, and reverse transcription polymerase chain reaction (RT-PCR) is currently regarded as the gold standard [45,46]. Given that this method has high sensitivity and specificity for viral nucleic acids, it can be used for serotyping, which is critical for epidemiological surveillance [8]. Multiplex RT-PCR platforms capable of simultaneously detecting dengue, chikungunya, and Zika would greatly assist with differential diagnosis in endemic settings. The *NS1* antigen detection test can detect more *NS1* glycoproteins produced during viral replication, in parallel with polymerase chain reaction (PCR). *NS1*-based immunoassays, such as rapid diagnostic tests (RDTs), help guide early patient triage but may differ in sensitivity across manufacturers [47,48]. As the infection progresses into the second week and viremia clears, the focus shifts to recognizing the host immune response. Detecting dengue-specific IgM provides evidence of recent infection, although cross-reactivity with other flaviviruses can occur [49,50]. IgG antibodies are absent during some stages of established disease, making their presence a better marker of prior exposure; however, confirming recent infection usually requires a fourfold increase in IgG titer between acute and convalescent serum samples, which is difficult in real-time clinical diagnosis [51,52]. The key characteristics of these methods are summarized in Table 1. Multiplex molecular diagnostic platforms capable of simultaneously detecting dengue, chikungunya, and Zika viruses may improve differential diagnosis and outbreak preparedness in endemic settings.

There remains an unequal capacity for diagnosing arboviral diseases in Saudi Arabia. Although reference laboratories in large cities such as Jazan and Makkah have PCR capacity, the paucity of molecular testing in rural clinics leads to delays in case confirmation and action [10,53]. With reliance on clinical suspicion and syndromic reporting, there will be underdiagnosis, especially of mild or atypical cases and of infections that mimic other arboviruses, including CHIKV and ZIKV [54,57,58]. Cross-reactivity among flaviviruses also confounds serologic testing, underscoring the importance of confirmatory assays such as plaque reduction neutralization tests. Consequently, it is time to invest in multiplex and differential diagnostics at the national laboratory level to improve the capacity to define arboviral circulation and enhance outbreak readiness. However, the backbone of prevention remains vector control.

## 6. Prevention and Vector-Control Programs

Because vaccines remain unavailable for many arboviral infections and vector exposure continues in densely populated urban settings, integrated vector-control strategies remain the primary approach for arboviral prevention in Saudi Arabia. The national strategy, led by the Ministry of Health in collaboration with local authorities, is formally based on the Integrated Vector Management strategy tool, which promotes an integrated approach that combines synergistic interventions to achieve sustainable control [59,60]. Current and potential strategies within this framework are summarized in Table 2. Chemical control remains a major component of current vector-control strategies and is pursued through a two-pronged approach. The control’s goal is to reduce mosquito numbers by eliminating their breeding places using methods such as temephos in liquid form (i.e., larviciding). Adult (life-stage) space spraying, fogging, or ultra-low-volume application of pyrethroid insecticides can quickly reduce the number of contaminated adults [61,62]. The prolonged reliance on pyrethroid-based interventions has contributed to the development of insecticide resistance in *Aedes aegypti* populations, particularly in dengue-endemic regions. This trend threatens the long-term effectiveness of conventional fogging-based vector-control programs. The rising prevalence of insecticide resistance across *Aedes aegypti* populations from Jazan at the southern end to downstream regions poses a threat to this essential tool; indeed, this overreliance on chemical control strategies is now seriously in question [13]. Future preparedness strategies should incorporate climate-informed predictive modeling as part of routine surveillance and outbreak preparedness. Geographic Information System (GIS)-based spatial models can identify transmission hotspots and support targeted vector-control interventions. Climate-suitability and remote sensing models may facilitate early warning systems by identifying periods and locations of elevated transmission risk. Mechanistic transmission models that integrate temperature, rainfall, humidity, vector abundance, and human mobility can support outbreak forecasting during high-risk periods, including Hajj and Umrah seasons. In parallel, genomic epidemiology and machine-learning approaches may strengthen outbreak tracking, serotype monitoring, and evidence-based allocation of diagnostic and public health resources.

Recognizing the limitations of chemical-only approaches, Saudi Arabia’s Integrated Vector Management strategy increasingly emphasizes environmental and biological control measures. Environmental management focuses on source reduction through eliminating mosquito breeding habitats, improving water storage practices, and enhancing municipal sanitation [70]. However, sustained implementation remains challenging because effective control depends heavily on long-term community participation and intersectoral coordination [73]. Biological control approaches are increasingly being explored as complementary components of integrated vector management. These include the use of larvivorous fish such as *Gambusia affinis* in suitable aquatic environments and the deployment of *Wolbachia*-infected *Aedes aegypti* populations to reduce vector competence for arboviruses [54,74]. Although these interventions have shown encouraging results in several endemic settings, additional studies are required to evaluate their ecological suitability, operational feasibility, community acceptance, and long-term effectiveness under local Saudi Arabian conditions. Prior to this, the Ministry of Health had only provided seasonal control of vector populations, resistance management, and public requests for cloud-based digital media spraying [60]. Such situations arise from human resource limitations and operational constraints. Resource limitations and operational constraints continue to hinder the implementation of decentralized vector control in some regions.

Community-based participatory programs and working group support for digital GIS data are intended to improve targeted interventions and increase the frequency of behavioral change, with the hope that such strategies will enhance sustainability; however, the challenge remains to find robust methods for ongoing intervention [71,72]. Only if entomological surveillance is strengthened, insecticides are used judiciously, community participation is increased, and new methods are expanded can IVM solidify into a firm foundation for launching arbovirus control efforts [63]. Operational implementation of a One Health framework in Saudi Arabia would require clearly defined institutional responsibilities. The Ministry of Health should coordinate human disease surveillance and outbreak response; municipal authorities should oversee sanitation, water infrastructure, and vector-source reduction; environmental agencies should support ecological monitoring and climate-risk assessment; and academic and research institutions should contribute genomic surveillance, predictive modeling, and operational research. Integration of these sectors through a centralized digital surveillance platform could improve real-time risk assessment and coordinated outbreak preparedness.

## 7. Cross-Border and Travel Implications

Because of Saudi Arabia’s geopolitical position and its role as one of the world’s largest annual destinations for religious pilgrimage, it occupies a unique epidemiological position due to its extensive international connectivity and annual influx of pilgrims from arbovirus-endemic regions. Millions of pilgrims travel annually to Makkah for Hajj and Umrah, many of whom originate from countries where arboviral diseases are endemic. Some countries from which pilgrims set off are highly endemic for diseases such as dengue fever [65]. With the increasing number of international travelers and pilgrims arriving in densely populated urban centers such as Makkah and Jeddah, where *Aedes aegypti* is well established, the risk of novel arboviral pathogens is rising. Imported infections may initiate local transmission when introduced into areas with established competent vectors. Mass gatherings may facilitate pathogen introduction and increase the complexity of infectious disease surveillance and response, thereby increasing opportunities for infectious disease transmission. Despite efforts to control vectors and educate the public, the possibility of introducing unknown arboviruses through imported infectious diseases will remain. One such outbreak might cross national borders, complicating global arbovirus dynamics. Movement between there and Yemen during the war, especially during periods of increased disease activity, can increase further risks of importing diseases like dengue fever [14,64]. Therefore, cross-border population mobility across the Saudi–Yemeni border could promote the transboundary dissemination of arboviruses. Inferred evolutionary relationships among dengue virus strains from molecular epidemiological studies indicate that closely related flavivirus lineages are endemic in several international networks across the region [27,66], suggesting that arboviral transmission on the Arabian Peninsula occurs within a highly networked landscape.

In addition, Saudi Arabia has a large expatriate community from South and Southeast Asia, which are areas of high endemicity for arboviral infections. Thus, there may be one further route for the virus into the Kingdom: international labor migration. Screening measures at points of entry, such as thermal screening, are less useful for arboviral infections because many infected individuals are either asymptomatic or present during the incubation period [75].

Addressing these challenges will require stronger surveillance at points of entry, additional surveillance at these points, and improvements to both syndromic and laboratory-based systems. In addition, the region needs knowledge management mechanisms to facilitate the sharing of country-level data for analysis. We focus our efforts on travelers in regions with a risk of illness; we conduct joint vector control with migrant and factory workers during mass gatherings; and we promote regional surveillance with our cooperation partners. Such cooperation between Saudi Arabia and countries in the region is advantageous to both long-term public health and efficiency savings. Without coordination, however, such efforts may cancel each other out. Only in a spirit of mutual support can they enhance regional preparedness and coordinated outbreak response for everyone involved. Saudi Arabia has just begun to delve into genomic surveillance, outbreak monitoring, and supranational data sharing. National preparedness efforts may remain limited without coordinated regional collaboration [67].

## 8. Research Gaps and Surveillance Strengthening

Despite increasing recognition of arboviral diseases as an emerging public health concern in Saudi Arabia, important gaps remain in surveillance, research, and preparedness. National surveillance systems may underestimate the true burden of infection because mild, asymptomatic, and atypical cases frequently remain undetected. Expanded seroprevalence studies are needed to better characterize population immunity, identify risk factors for transmission, and inform evidence-based prevention strategies. Climate-informed disease modeling has considerable potential to strengthen proactive outbreak preparedness in Saudi Arabia. Integrating meteorological, entomological, urbanization, and epidemiological data into predictive frameworks may support early warning systems, optimize vector-control timing, and improve resource allocation in high-risk regions. National predictive models could further guide targeted public health interventions by enabling scenario forecasting under different climate and transmission conditions. These approaches may improve preparedness planning and enhance vector-control efficiency. Saudi Arabia must urgently invest in strengthening laboratory and genomic surveillance. Expanded implementation of multiplex molecular diagnostic platforms would improve case detection, differential diagnosis, and outbreak surveillance. Genomic sequencing diagnostics shed light on virus evolution and transmission, enabling prompt detection of outbreaks and the development of measures. Future research should prioritize spatiotemporal disease modeling, climate suitability mapping, genomic epidemiology, remote sensing surveillance, and machine learning approaches that integrate meteorological, entomological, and epidemiological datasets to improve outbreak prediction and preparedness.

The level of community engagement remains low. Knowledge, Attitudes, and Practices surveys can reveal how community members view vector-borne diseases, which is critical for developing culturally sensitive public health measures. Filling the gaps in research and surveillance requires a proactive One Health approach that integrates human, environmental, and vector data to enable real-time risk assessment and coordinated action against arboviral threats. This is essential for Saudi Arabia’s outbreak response. Routine genomic surveillance could improve the detection of viral introductions, monitor serotype replacement, identify transmission networks, and strengthen outbreak-tracking capabilities.

## 9. Expert Opinion and Future Perspective

Current epidemiological and environmental trends suggest that Saudi Arabia may face an increasing risk of arboviral disease emergence and expansion over the coming decades. The epidemiological situation in Jazan represents an important sentinel indicator, but a sentinel event that highlights significant weaknesses in current preparedness systems. Current reliance on insecticide fogging may become less effective as insecticide resistance increases among vector populations. To prevent more frequent and severe outbreaks and to enable the subsequent establishment of CHIKV or ZIKV, a profound shift to three core pillars is required. First, predictive, data-driven public health is grounded in integrating climate models, real-time meteorological data, and satellite-derived urbanization measures into entomological surveillance to produce accurate dengue risk forecasts that support pre-outbreak interventions, such as targeted larviciding and community clean-ups. Second, the operationalization of the One Health doctrine, which requires strengthening intersectoral coordination and promoting collaboration among city managers, public health leaders, and urban planners to improve waste management, ensure resilient water systems, reduce household water storage, and accelerate the development and field testing of new biological tools, including *Wolbachia*-infected mosquitoes. Third, regional collaboration and regional public health authorities should recognize that a purely national response is insufficient in a connected world. Enhancing Yemen’s surveillance and response capabilities is not only an ethical but also a cost-effective pathway to long-term regional health protection. In contrast, digital health initiatives such as a pre-travel application for Hajj and Umrah pilgrims’ health screening could significantly enhance public awareness, enable real-time symptom monitoring, and prompt reporting.

## 10. Conclusions

Arboviral diseases, particularly dengue, are an emerging public health problem in Saudi Arabia, driven by the combined effects of climate change, rapid urbanization, increased human mobility, and the widespread distribution of *Aedes aegypti*. Evidence from endemic regions, such as Jazan, Makkah, and Jeddah, indicates that ecological suitability, population density, and mass gatherings all create conditions conducive to sustained vector-borne disease transmission. Although dengue is the predominant arboviral infection currently documented in the Kingdom, the ecological and epidemiological conditions that favor transmission also indicate a potential future risk of introduction or localized emergence of other arboviruses, including chikungunya and Zika viruses.

The existing surveillance and vector-control measures need to be complemented with integrated, climate-informed, and data-driven approaches. Investments in multiplex molecular diagnostics, genomic surveillance, insecticide-resistance monitoring, GIS-based risk mapping, and predictive modeling will be critical for enhanced outbreak preparedness and early detection. Equally important is the deployment of sustainable community-based vector-control programs supported by improved water infrastructure, environmental management, and public health education.

A practical way to coordinate preparedness and rapid response is to implement a One Health framework that integrates human, vector, environmental, and climate surveillance systems. Because the Kingdom is a major hub for international travel, labor migration, and religious mass gatherings, regional collaboration and cross-border surveillance will be essential components of long-term prevention of arboviral diseases. Continued investment in research, surveillance infrastructure, and intersectoral collaboration will bolster Saudi Arabia’s capacity to anticipate, prevent, and respond effectively to emerging arboviral threats amid accelerating environmental change.

## Figures and Tables

**Table 1:** Diagnostic approaches for arboviral infections: targets, optimal testing windows, advantages, and limitations. Here, we summarize the main molecular laboratory techniques for diagnosing arbovirus infections, such as dengue, including their targets, optimal utilization periods (windows), advantages, and limitations. Test selection varies based on symptom duration and specific diagnostic or surveillance considerations.

Method	Target	Optimal Timing (Post-Symptom Onset)	Advantages	Limitations	References
RT-PCR	Viral RNA	Days 1–5 post-symptom onset (Acute phase)	High sensitivity & specificity; identifies specific serotype.	Requires specialized laboratory infrastructure and trained personnel and has a short detection window.	[51,52]
NS1 Antigen Test	Viral *NS1* protein	Days 1–5 post-symptom onset (Acute phase)	Rapid diagnostic tests (RDTs) provide early antigen detection during acute infection.	Variable sensitivity between products/strains; shorter window than PCR.	[53]
IgM Serology	Host IgM antibodies	≥5 days post-symptom onset (Convalescent phase)	Indicates recent infection; useful after viremia clears.	Potential serological cross-reactivity with other flaviviruses; false negatives early in infection.	[54,55]
IgG Serology	Host IgG antibodies	≥14 days post-symptom onset; paired acute and convalescent samples	Indicates past exposure; confirms recent infection with paired samples.	Limited utility for acute diagnosis using a single sample; potential cross-reactivity	[56]

RT-PCR, reverse transcription polymerase chain reaction; NS1, non-structural protein 1; IgM, immunoglobulin M; IgG, immunoglobulin G; RDTs, rapid diagnostic tests.

**Table 2:** Integrated vector management strategies for arboviral prevention and control in Saudi Arabia. The table summarizes the current implementation status, operational challenges, and strategic recommendations to strengthen vector control within a One Health framework.

Category	Method/Tool	Current Implementation Status in Saudi Arabia	Operational Challenges	Recommended Strategies	References
Chemical Control	Larviciding (e.g., temephos, pyriproxyfen)	Widely implemented in urban dengue-endemic areas such as Jazan and Makkah	Development of insecticide resistance; need for repeated applications; operational costs	Establish routine resistance monitoring programs; rotate insecticide classes; integrate with environmental management	[63]
	Adulticiding (ULV fogging with pyrethroids)	Primary emergency response during outbreaks	Short-term effectiveness; insecticide resistance; limited penetration into indoor breeding sites	Primarily recommended for targeted outbreak response; apply targeted spraying based on entomological surveillance	[13]
Environmental Management	Source reduction (elimination of breeding sites)	Promoted through municipal sanitation campaigns	Requires sustained community participation; difficulty accessing private households	Strengthen community-based vector-control programs; improve waste management and water storage practices	[64,65]
	Improved water storage infrastructure	Limited implementation in some urban areas	Intermittent water supply encourages household water storage	Promote sealed water tanks and improved household water infrastructure	[42]
Biological Control	Larvivorous fish (*Gambusia affinis*)	Limited pilot implementation in reservoirs and ornamental water bodies	Limited applicability in small containers; ecological concerns	Evaluate ecological suitability and integrate it into targeted vector-control programs	[66]
	*Wolbachia*-infected mosquitoes	Pilot or experimental implementation stage	Requires regulatory approval, community acceptance, and field validation	Conduct pilot trials in dengue-endemic regions; evaluate long-term effectiveness	[67]
Technological Surveillance	GIS-based spatial risk mapping, climate-informed surveillance, and predictive modeling	Emerging but not fully integrated into routine surveillance	Limited integration between meteorological, entomological, and epidemiological data	Develop a unified digital platform integrating climate, vector, and disease surveillance data	[68]
	Climate-based early warning systems	Not yet widely implemented	Lack of real-time climate–health data integration	Integrate meteorological forecasting with dengue surveillance systems to guide early interventions	[39]
Community Engagement	Knowledge–Attitude–Practice (KAP) surveys	Sporadic implementation in research settings	Limited translation of findings into policy	Conduct periodic KAP surveys to inform culturally appropriate public health messaging	[69]
	Community-led vector-control campaigns	Underutilized	Limited sustained community engagement	Promote participatory approaches involving schools, religious institutions, and local leaders	[69,70]
Digital & One Health Approaches	Integrated One Health surveillance	Limited implementation	Sectoral fragmentation between health, environment, and municipal authorities	Develop integrated surveillance platforms linking human, entomological, environmental, and climate data across public health, municipal, and environmental sectors within a One Health framework.	[71,72]
	Mobile reporting and citizen surveillance apps	Limited implementation	Limited digital surveillance infrastructure	Develop mobile apps for reporting mosquito breeding sites and dengue symptoms	[71,72]
Genomic Surveillance	Viral sequencing and molecular epidemiology	Limited implementation	Limited sequencing capacity and data integration	Expand genomic surveillance for outbreak tracking and serotype monitoring	[66,73]

GIS, Geographic Information System; ULV, ultra-low volume.

## Data Availability

Not applicable.
